# Sicherstellung der hausärztlichen Versorgung in Deutschland – Befunde einer quantitativen Befragung von Allgemeinmediziner*innen

**DOI:** 10.1007/s00103-024-03896-4

**Published:** 2024-06-11

**Authors:** Julian Wangler, Michael Jansky

**Affiliations:** Zentrum für Allgemeinmedizin und Geriatrie, Universitätsmedizin Mainz, Am Pulverturm 13, 55131 Mainz, Deutschland

**Keywords:** Hausärztemangel, Landarzt, Landpraxis, Primärversorgung, Gewohnte Versorgung, GP shortage, Rural physician, Country practice, Primary care, Established care

## Abstract

**Hintergrund:**

Angesichts der Gefahr einer Verknappung niedergelassener Allgemeinmediziner*innen stellt sich die Frage, welche Konzepte wirksame Beiträge leisten können, um eine drohende Mangelversorgung abzuwenden. Bis dato fehlt es an Studien, die beleuchten, wie Hausärzt*innen aus ihrer beruflichen Erfahrung zu verschiedenen Ansätzen zur langfristigen Sicherstellung der Primärversorgung stehen.

**Ziel der Arbeit:**

Ziel der Studie war es, Positionen, Haltungen und Erfahrungswerte von Hausärzt*innen mit Blick auf eine Sicherstellung der allgemeinmedizinischen Breitenversorgung zu ermitteln.

**Methoden:**

Im Zuge einer Online-Befragung wurden zwischen Februar und Juni 2023 insgesamt 4176 Hausärzt*innen befragt. Über die deskriptive Analyse hinaus erfolgte zur Ermittlung von signifikanten Unterschieden zwischen 2 Gruppen ein t‑Test bei unabhängigen Stichproben.

**Ergebnisse:**

42 % beobachten im Umfeld einen merklichen Schwund allgemeinärztlicher Praxen. 53 % bescheinigen der Hausarztmedizin eine sinkende Attraktivität für den ärztlichen Nachwuchs, was 3 Problembereichen zugeordnet wird: 1) Stellung der hausärztlichen Versorgung im Gesundheitswesen, 2) Voraussetzungen in Aus- und Weiterbildung, 3) Arbeitsbedingungen. Zur Sicherung der Hausarztmedizin sprechen sich die Befragten v. a. für folgende Ansatzpunkte aus: Einrichtung eines Primärarztsystems (85 %), vermehrte Förderung von Interesse und Berührungspunkten in Aus- und Weiterbildung (80 %), Stärkung multiprofessioneller ambulanter Versorgungszentren (64 %), Restrukturierung von Curricula (56 %) und Zulassungskriterien zum Medizinstudium (50 %), Reform der allgemeinmedizinischen Weiterbildung (53 %).

**Diskussion:**

Hausärzt*innen haben eigene Vorschläge und Präferenzen, die Expertisen und Sachverständige ergänzen. Bei der Planung, Implementierung und Evaluation von Maßnahmen zur Stabilisierung der hausärztlichen Versorgung sollten Allgemeinmediziner*innen konsequenter als bislang involviert werden.

**Zusatzmaterial online:**

Zusätzliche Informationen sind in der Online-Version dieses Artikels (10.1007/s00103-024-03896-4) enthalten.

## Abstract (en)

**Background:**

Given the risk of a shortage of general practitioners in private practice, the question arises as to which concepts can make an effective contribution. To date, there is a lack of studies that comprehensively shed light on how general practitioners, based on their professional experience, view different approaches to ensuring primary care in the long term.

**Objectives:**

The aim of the study was to determine the positions, attitudes, and experiences of general practitioners with regard to ensuring primary care.

**Methods:**

Using an online survey, a total of 4176 general practitioners were surveyed between February and June 2023. In addition to the descriptive analysis, a t-test on independent samples was used to determine significant differences between two groups.

**Results:**

Of those surveyed, 42% reported a noticeable decline of general medical practices in their area. In addition, 53% saw a declining attractiveness of primary care for young doctors, which is attributed to three problem areas: 1) the position of primary care in the healthcare system, 2) requirements for training and further education, and 3) working conditions. In order to secure primary care, those surveyed were primarily in favor of the following approaches: establishing a primary care physician system (85%), increasing the promotion of interest and points of contact in training and further education (80%), strengthening multi-professional outpatient care centers (64%), restructuring curricula (56%) and admission criteria for medical studies (50%), and reforming general medical training (53%).

**Conclusions:**

As the results show, general practitioners have their own suggestions and preferences that complement existing expert assessments. General practitioners should be more consistently involved in the planning, implementation, and evaluation of measures to stabilize primary care.

## Einleitung

Seit einigen Jahren befasst sich die Gesundheitspolitik in Deutschland verstärkt mit dem Szenario eines drohenden Mangels hausärztlicher Versorger*innen [1]. Blickt man auf die soziodemografische Situation, so zeigt sich, dass inzwischen jede/r dritte Hausärzt*in ein Alter von 60 Jahren oder mehr erreicht hat [2, 3]. Die Statistik der Kassenärztlichen Bundesvereinigung (KBV) weist für das Jahr 2021 ca. 4000 offene Stellen für Hausärzt*innen aus, wobei im Durchschnitt der vergangenen Jahre der Anteil der vakant gebliebenen KV-Sitze bei ca. 60 % liegt [2].

Berechnungen zeigen, dass jährlich etwa 1700 Hausärzt*innen aus der Berufstätigkeit ausscheiden, während die fachärztlichen Anerkennungen zwischen 1300 und 1400 liegen [4, 5]. Obwohl Letztere zuletzt wieder anstiegen, ist der Kompensationsbedarf zur Aufrechterhaltung der bestehenden Versorgungskapazitäten seit geraumer Zeit ungedeckt. Verschärft wird diese Entwicklung dadurch, dass nachrückende Hausärzt*innen eine größere Präferenz für Teilzeit- und flexiblere Arbeitsmodelle sowie eine Festanstellung zeigen [6, 7]. Infolge dieser zusammenwirkenden Trends ist bis zum Jahr 2030 ein Defizit von bis zu 23.000 hausärztlichen Vollzeitäquivalenten möglich, wobei hiervon besonders ländliche und strukturschwache Regionen betroffen sein dürften [8]. Eine Studie der Robert Bosch Stiftung aus dem Jahr 2021 prognostizierte bis 2035 für den ungünstigsten Fall eine Unterversorgung von 40 % aller deutschen Landkreise [5, 9].

Vor dem Hintergrund solcher Befundlagen und Projektionen wird fortdauernd über geeignete Maßnahmen diskutiert, um einem zu starken Schwund allgemeinmedizinischer Behandler*innen möglichst effektiv entgegenzuwirken [10]. Ein konzeptioneller Schwerpunkt zur strukturellen Stärkung der Primärversorgung zielt auf die breitflächige Schaffung multiprofessioneller ambulanter Zentren, die einerseits den Rückgang traditioneller Einzelpraxen kompensieren und andererseits die multiprofessionelle Anbindung der Hausarztmedizin verbessern sollen [11]. Ebenfalls von diversen Akteuren gefordert wird die Einführung eines Primärarztsystems, um die hausärztliche Rolle spürbar aufzuwerten [12, 13]. Ein weiterer Ansatzpunkt, der bereits seit Jahren kontinuierlich verfolgt wird, betrifft die Schaffung neuer Delegationsmodelle innerhalb von hausärztlichen Praxen, die Allgemeinmediziner*innen entlasten und zur Versorgungseffizienz beitragen sollen [14].

Um die Ansiedlung von Hausärzt*innen insbesondere in ländlichen Regionen zu fördern, werden mehrere Strategien verfolgt. Beispiele sind eine stärkere Bedarfsplanung mit regionaler Verteilungswirkung [15], aber auch eine wirksamere Anreizstruktur mit Niederlassungsprämien oder Stipendien. Ebenfalls angeregt wird eine (stärkere) Strukturierung der Weiterbildung durch Landkreise und Kommunen, aber auch kassenärztliche Vereinigungen, Verbände, Institute für Allgemeinmedizin und Krankenkassen [16]. Um das Reservoir für die Rekrutierung von hausärztlich tätigen Mediziner*innen auszuweiten, sind Vorschläge unterbreitet worden, den Anteil der Allgemeinmedizin an der Weiterbildung zu erhöhen, den Zugang zur Spezialist*innenweiterbildung zu quotieren sowie die hausärztliche Tätigkeit unter bestimmten Voraussetzungen stärker für einen Quereinstieg aus anderen Disziplinen zu öffnen [13, 17].

Neben der erfolgten Einführung von bundeslandspezifischen Landarztquoten gibt es weitreichende Vorschläge zur Anpassung der Aus- und Weiterbildung. Diese fokussieren einerseits eine curriculare Neugestaltung des Studiums der Humanmedizin, andererseits eine Modifikation von Zulassungskriterien, um eine hausärztliche Berufsperspektive zu befördern [18]. Nicht zuletzt sind Empfehlungen ausgesprochen worden, Art und Dauer der fachärztlichen Weiterbildung zu verändern und eine stärkere Ausrichtung an einer allgemeinärztlich niedergelassenen Tätigkeit vorzunehmen [19].

In Anbetracht der zahlreichen Akteure aus den Bereichen der Gesundheitspolitik und Versorgungsforschung, die sich zur Thematik äußern, ist auffällig, dass die genuin hausärztliche Perspektive bis dato vernachlässigt wurde. So gibt es nur wenig Evidenz, wie Hausärzt*innen aus ihrer beruflichen Erfahrung zu verschiedenen Ansätzen zur langfristigen Sicherstellung der Primärversorgung stehen. Es erscheint sinnvoll, bei der Planung, Umsetzung und Justierung solcher Schritte die Sichtweisen und Einschätzungen von Hausärzt*innen zu berücksichtigen, um praxisorientierte und nachhaltige Lösungen zu ergreifen.

Ziel der Studie war es, Positionen, Haltungen und Erfahrungswerte von Hausärzt*innen mit Blick auf eine langfristige Sicherstellung der allgemeinmedizinischen Versorgung einzuholen. Im Zentrum standen dabei v. a. folgende Fragestellungen:

- Wie wird die Zukunft der hausärztlichen Versorgung wahrgenommen?
- Inwiefern wird die hausärztliche Versorgung als gesichert angesehen?
- Welche Maßnahmen werden als vielversprechend und vordringlich erachtet, um die hausärztliche Versorgung sicherzustellen?

## Methoden

Im Frühjahr 2023 wurde eine Vollbefragung von Hausärzt*innen in 4 Bundesländern durchgeführt. Diese war ausgestaltet als Onlinebefragung mit schriftlich-postalischem Anschreiben.

### Erhebungsinstrument

Die Konzeption des Befragungsinstruments für die quantitative Querschnittsstudie (siehe Onlinematerial) erfolgte maßgeblich auf Grundlage einer qualitativen Vorstudie, bei der im Jahr 2022 insgesamt 96 hausärztlich tätige Mediziner*innen zur Thematik befragt wurden [20]. Daneben floss eine Literaturrecherche zum Thema in den Entwicklungsprozess ein (u. a. [4, 6, 13, 21, 22]). Insbesondere wurde auf die Arbeit von van den Bussche [13] zurückgegriffen, der Problematiken der gebündelten Sicherstellung der Primärversorgung diskutiert. Auf dieser Grundlage konnte eine Liste der abzufragenden Maßnahmen erstellt werden. Auch wurden 2 Fragen mithilfe des MLP-Gesundheitsreports 2022 adaptiert [23].

Der letztendliche Fragebogen beinhaltet insgesamt 23 Fragen und setzt sich aus 4 inhaltlichen Schwerpunkten zusammen:

- Status quo und Entwicklung der hausärztlichen Versorgung in längerfristiger Perspektive,
- persönliche Belastungserfahrungen sowie Beobachtungen zum Ärzt*innenmangel,
- favorisierte Ansätze bzw. Maßnahmen zur Sicherstellung der Hausarztmedizin,
- Beurteilung ergriffener Maßnahmen zur Sicherstellung und weitere Optimierungsansätze.

Neben den standardisierten Fragen wurden mehrere offene Fragen eingesetzt, um dem explorativen Charakter der Studie Rechnung zu tragen (Frage 4, 6, 9, 11, 16, 18, 20, 21).

Als soziodemografische Merkmale wurden Geschlecht, Alter, Praxisumgebung, Praxisform und Anzahl der Patient*innen pro Quartal erhoben. Vor dem Feldeinsatz wurde ein Pretest durchgeführt. Hierzu wurde der Fragebogen 50 zufällig ausgewählten Hausärzt*innen aus dem Umfeld der hausärztlichen Lehrbeauftragten der Abteilung Allgemeinmedizin vorgelegt. Der Pretest zeigte, dass eine gute Verständlichkeit und Strukturierung sowie Vollständigkeit der Antwortkategorien gegeben sind.

### Rekrutierung und Stichprobe

Auf schriftlich-postalischem Weg zur Teilnahme an der anonymisierten Befragung eingeladen wurden zwischen Februar und Juni 2023 sämtliche 13.913 als Behandler*innen aktive Hausärzt*innen in Baden-Württemberg (6664), Hessen (3839), Rheinland-Pfalz (2667) und im Saarland (743). Die Entscheidung zugunsten dieser Bundesländer erfolgte zum einen aus der Erwägung heraus, dass bevölkerungsstarke Flächenländer in die Studie eingeschlossen werden sollten, die zumindest eine angenäherte Repräsentanz der Hausärzteschaft in Deutschland haben. Zum anderen lagen den Autoren aufgrund kontinuierlicher Beforschung der hausärztlichen Versorgung für besagte Bundesländer aktuelle, vollständige Kontaktlisten vor. Es handelte sich um ein einmaliges Anschreiben, in dem die zu befragenden Ärzt*innen u. a. einen passwortgeschützten Zugang zur Onlinebefragung mitgeteilt bekamen (keine Incentives).

Der Rücklauf der Fragebögen betrug 30 %. Von den 4259 bearbeiteten Fragebögen waren 4176 vollständig ausgefüllt und gingen in die Auswertung ein. Tab. 1 stellt die gewonnene Stichprobe und repräsentative KV-Daten zum Aufbau der Hausärzteschaft in Deutschland gegenüber.

**Tab. 1:** Stichprobe in Gegenüberstellung mit Repräsentativstatistik

	Stichprobe (*N* = 4176)	Repräsentativstatistik
Geschlecht:	62 % männlich, 38 % weiblich	58 % männlich, 42 % weiblich^1^
Durchschnittsalter:	50 (Median: 51)	56 (Median: 57)^1^
Praxisumgebung:	49 % mittel- und großstädtisch, 51 % ländlich-kleinstädtisch	41 % mittel- und großstädtisch, 59 % ländlich-kleinstädtisch^1^
Praxisform:	55 % Einzelpraxen, 34 % Gemeinschaftspraxen, 11 % MVZ/sonstige Einrichtung	56 % Einzelpraxen, 38 % Gemeinschaftspraxen, 6 % MVZ/sonstige Einrichtung^2^
Patienten pro Quartal:	27 % 500–1500, 32 % 1501–2000, 41 % > 2000	Keine vollständigen Daten verfügbar
Akademische Lehrärzt*innen:	10 %	Keine vollständigen Daten verfügbar

^1^Basierend auf den KV-Versorgungsforschungsdaten für Rheinland-Pfalz (Stand: 31.12.2021),

abrufbar unter: https://www.kv-rlp.de/institution/engagement/versorgungsforschung/

^2^Basierend auf den KV-Versorgungsforschungsdaten für Deutschland (Stand: 31.12.2021),

abrufbar unter: https://www.kbv.de/html/gesundheitsdaten.php

### Datenanalyse

Die Daten wurden mittels SPSS 23.0 (IBM, Armonk, NY, USA) ausgewertet. Zur Ermittlung von signifikanten Unterschieden zwischen 2 Gruppen erfolgte ein t‑Test bei unabhängigen Stichproben. Es wurden 2 Signifikanzniveaus getestet (mittlere Differenz bei *p* < 0,05 und *p* < 0,001). Diese parametrische Methode weist eine hohe Teststärke auf und gilt als statistisch robust. Mit der Fallzahl, der Normalverteilung der zu unterscheidenden Gruppen und der Tatsache, dass die Stichproben aus derselben Grundgesamtheit stammen, waren die notwendigen Voraussetzungen gegeben [24].

Die Auswertung der offenen Fragen basiert auf einer Nachcodierung im Sinne der qualitativen Inhaltsanalyse. Dies beinhaltete für die Freitextantworten zu jeder offenen Frage die Erstellung eines basalen Kategoriensystems [25]. Als Reporting Statement wurde STROBE herangezogen. Prägnante Freitextantworten, die Teil der Ergebnisdarstellung sind, finden sich gesammelt in Tab. 2 (im Folgenden wird anhand der Zitatnummer (Zx) auf die Zitate in Tab. 2 verwiesen).

**Tab. 2:** Ausgewählte Zitate, Freitextantworten (Fragen 4, 6, 9, 11, 16, 18, 20, 21)

Zitat-Nr.	Zitat
1	„Aus irgendeinem Grund haben wir zu lange blind darauf gesetzt, dass schon ein Teil der Studierenden nach dem Studium automatisch Hausarzt werden – eine falsche Annahme, die uns jetzt teuer zu stehen kommt.“
2	„Wir beobachten seit geraumer Zeit, dass uns die Hausärzte auszugehen drohen, weil zu wenig in das System nachkommt. … Wir haben bereits lange kein Erkenntnisdefizit, sondern ein Handlungsdefizit. Die Hebel zur Anpassung des Systems wurden nicht rechtzeitig umgelegt.“
3	„Ich sehe die reale Gefahr eines äußerst gefährlichen Teufelskreises, an dessen Ende die Substanz der Hausärzteschaft zerbröselt.“
4	„Junge Mediziner gehen nicht aus dem Grund nicht mehr in die Hausarztpraxen weil sie zu wenig Geld verdienen, sondern weil die Rahmenbedingungen als zu unattraktiv gelten.“
5	„Das Berufsbild des Hausarztes ist vermutlich wichtiger und gefragter denn je. Um diesen Bedarf einzulösen, bedarf es jedoch tiefgreifender Weichenstellungen. … Es fehlt an diesem generellen Umdenken.“
6	„Hausärzte müssen im Hinblick auf ihre Entscheidungskompetenz gestärkt werden. Nach Lage der Dinge ist der Schlüssel dazu nur ein Primärarztsystem, das seinen Namen verdient.“
7	„Kombinierte Zentren leisten zwar einer Verlagerung weg von der traditionellen Praxis Vorschub, was man ambivalent sehen kann. Es gibt jedoch eine große Chance, Hausärzte von ihrem Einzelkämpferdasein zu befreien, den Bereich zu modernisieren und besser an andere Berufsgruppen anzubinden.“
8	„Das Prinzip ist sinnvoll: Die Ärzte dort arbeiten auf eigene Rechnung, sie teilen sich aber auch bestimmte Kosten, Teamarbeit wird großgeschrieben. … Meine eigene Tochter arbeitet in einem solchen Ärztezentrum und könnte sich nicht mehr vorstellen, in eine normale Praxis zu gehen.“
9	„Ich bin alles andere als überzeugt, dass mehr Studienplätze automatisch zu mehr Ärzten führen. Das System ist so voreingestellt, dass wir in erster Linie Spezialisten heranzüchten. Genau das muss geändert werden.“
10	„Das Denken, dass alles am Hausarzt hängt, muss sich noch stärker ändern. Hier gibt es noch große Potenziale. Dazu gilt es, nicht-ärztliche Berufe aufzuwerten, an die eine Delegation erfolgen kann. Dieses Prinzip funktioniert in anderen Ländern sehr viel besser als bei uns.“
11	„Die Probleme werden sich verschärfen, wenn wir nicht stärker in das ganze Verteilungssystem eingreifen. Die Datenlage, wie groß der Bedarf an verschiedenen Disziplinen ist, müssen hierzu ausschlaggebend sein. Das hat zur Folge, dass die Allgemeinmedizin einen größeren, festgeschriebenen Anteil im Bereich der Weiterbildung erhält und dass notfalls auch die freie Berufswahl ein Stück weit hinter der Priorität des Bedarfs zurückzustecken hat.“
12	„Ich habe es ja aus eigener Erfahrung als Lehrarzt miterlebt: So ein Begleitprogramm öffnet Horizonte; es führt sorgsam heran und motiviert. … Es vermittelt, was die breit gefächerte Arbeit des Allgemeinarztes praktisch bedeutet. … Dabei lassen sich Hausärzte sehr sinnvoll einbeziehen. Meines Erachtens weisen solche Modelle in die Zukunft.“
13	„Die Bemühungen der letzten Jahre sind grundsätzlich als richtig zu bewerten, trotzdem sind sie lange nicht beherzt und entschlossen genug, wenn man zugrunde legt, welches Ausmaß des Ärztemangels in den nächsten Jahren auf uns zukommen wird. … Es ist eine reale Gefahr, dass wir den Anschluss an diese Entwicklung verpassen und dann wieder einmal der berüchtigten Welle hinterherlaufen.“
14	„Es wird derzeit eine Menge herumlaboriert, aber das Problem bleibt, dass die Hausarztmedizin nach wie vor nicht attraktiv genug ist, um ausreichend neues Personal anzuziehen.“
15	„Es ist immer noch nicht geschehen: … Die Studierenden müssen in einem umfassenden Sinne viel stärker an die Hausarztmedizin herangeführt werden. Darunter sind nicht in erster Linie irgendwelche Quoten zu verstehen, sondern Einblicke, Motivation und Kompetenzen. Dahingehend haben wir nach wie vor eine große Leerstelle.“
16	„Die Allgemeinmedizin speziell und der niedergelassene Bereich allgemein muss ins Zentrum des Studienstoffes rücken – mit allem, was dazu gehört.“
17	„Diese Quotendiskussionen und Quotenlösungen kennen wir nun hinlänglich aus anderen Bereichen. Dort sind Quoten selten die Lösungen, sondern verstärken eher das Bild, dass irgendjemand Nachhilfe nötig hat, weil er besonders schlecht da steht.“
18	„Ein angehender Hausarzt oder Hausärztin ist aus speziellem Holz geschnitzt. Wir müssen da besser hinsehen, um die Richtigen zu gewinnen und diese frühzeitig zu fördern. … Dies müssen nicht die Besten und die Klügsten mit den tollsten Abiturnoten sein, sondern gerade Hausärzte sind qua Profession Leute mit Lebens- und Menschenerfahrung. … Das System muss Persönlichkeit und Vorerfahrungen noch mehr honorieren.“
19	„Bei der Weiterbildung hapert es an verschiedenen Stellen. Sie ist nicht kompakt genug, aber auch nicht nah genug an der hausärztlichen Realität dran. Kompetenzen werden nicht praxisnah genug vermittelt, aber auch zu wenig aktuelles Wissen wie Evidenznähe oder digitale Möglichkeiten der Medizin.“
20	„Die Idee, dass die Universitäten auch in der Weiterbildungszeit mit die wichtigsten Partner bleiben, ist sehr wichtig und trägt zur Professionalisierung bei.“
21	„Diese Einrichtungen können entscheidend dazu beitragen, dass die Vorbereitung und Begeisterung für den Job besser gelingt und dass auch Abbruchquoten in der Weiterbildung sinken. … Sie müssen noch mehr Förderung und mehr Effektivität erhalten.“
22	„Ich wünsche mir, dass wir Hausärzte nicht mehr am Gängelband von Bürokratie und Kostenvorgaben hängen. … Mit einer permanenten Regressdrohung behandelt es sich höchst unbequem. … Ebenso wünsche ich mir, dass eine bessere Steuerung der Gesundheitsversorgung dazu führt, dass wir nicht mehr die Ausputzer von Fehlstellungen sind, für die wir nichts können. … Dann kann auch ein anderes Bild von uns in der Öffentlichkeit entstehen.“
23	„In einem sich immer weiter aufspaltenden und verästelnden Gesundheitssystem gibt es einen enormen Bedarf nach kompetenten Allroundern. In meinen Augen führt kein Weg daran vorbei, die Hausarztmedizin in eine neue Ära zu bringen.“
24	„Wenn wir ganz unterschiedliche Stellschrauben nutzen, um Hausarztpraxis umfassend zu modernisieren, zu entlasten und aufzuwerten, dann kann sie wieder das sein, was sie früher einmal war: das Rückgrat des Versorgungsgeschehens. … Es gibt jedoch eine Menge zu tun.“

## Ergebnisse

### Status quo und Entwicklung der hausärztlichen Versorgung in längerfristiger Perspektive

23 % der Befragten gehen davon aus, die ambulante Gesundheitsversorgung werde sich in den kommenden Jahren verbessern (11 %) oder gleichbleiben (12 %), während 67 % eine moderate (31 %) oder sogar deutliche (36 %) Verschlechterung erwarten. Analog dazu halten lediglich 20 % die hausärztliche Versorgung, wie sie heute besteht, für die kommenden 1–2 Jahrzehnte gesichert; 77 % gehen von einer weniger guten (33 %) oder gar gänzlich ungesicherten (44 %) Situation aus. Geht es um Zukunft und Entwicklungsperspektiven der ambulanten Primärversorgung zeigen sich 24 % sehr (3 %) oder eher (21 %) zuversichtlich, während 73 % eher (44 %) oder sehr besorgt (29 %) sind (Z1, Z2).

Eine Aufschlüsselung der oben angesprochenen Fragen belegt, dass Ärzt*innen in Kleinstädten und Landgemeinden eine deutlich pessimistischere Einschätzung der Perspektive der Hausarztmedizin haben als Ärzt*innen in Groß- und Mittelstädten (Tab. 3).

**Tab. 3:** Einschätzungen zur Sicherung der ambulanten und hausärztlichen Gesundheitsversorgung

Fragen 1, 2, 8 und 10 (*N* = 4176)	Gesamtzustimmung (%)	Urbane vs. Landärzt*innen (%)
**Frage:** *Wie schätzen Sie das ein: Wird sich die Gesundheitsversorgung in Deutschland in den nächsten Jahren insgesamt eher verbessern oder eher verschlechtern?*	56 % (Eher verschlechtern/ Deutlich verschlechtern)	51/61
**Frage:** Wie schätzen Sie dies für speziell für die ambulante Versorgung ein, also niedergelassene Haus- und Fachärzt*innen? Wird sich die Gesundheitsversorgung hier …	67 % (Eher verschlechtern/ Deutlich verschlechtern)	53/81*
**Frage:** *Wenn es um Zukunft und Entwicklungsperspektiven der hausärztlichen Versorgung im Zeitraum der nächsten 10 bis 20 Jahre geht: Sind Sie diesbezüglich eher zuversichtlich oder eher besorgt?*	73 % (Eher besorgt/ Sehr besorgt)	60/86*
**Frage:** *Wenn es um die längerfristige Gewährleistung der hausärztlichen Versorgung geht. Wie schätzen Sie dies ein: Wie gut ist die hausärztliche Versorgung in Deutschland für die kommenden Jahrzehnte gesichert?*	77 % (Eher nicht so gut gesichert/ Überhaupt nicht gesichert)	65/89*

Signifikanter Unterschied: *p < 0,001

Bezogen auf die gesamte Stichprobe nehmen 30 % aller Befragten an, dass sich in den kommenden 10–20 Jahren ein (verstärkter) Mangel an Hausärzt*innen vorwiegend in ländlichen und strukturschwachen Regionen manifestieren wird. 37 % gehen darüber hinaus von einem breitflächigen Mangel allgemeinärztlicher Versorger*innen auch in städtischen Einzugsgebieten aus (16 % kein nennenswerter Mangel). Unter denjenigen Befragten, die von einem Mangel an Hausärzt*innen ausgehen, rechnen 27 % mit einem Versorgungsdefizit zwischen 5 % und 15 %; 57 % nehmen einen ungedeckten Bedarf von 15 % und mehr an.

Eine kombinierte Auswertung zweier offener Fragen (9, 11) zeigt, dass viele Befragte zum einen eine in erheblichen Teilen Deutschlands anzutreffende Unterversorgung antizipieren, die wiederum Auswirkungen auf eine funktionierende Zuleitung zu anderen Versorgungsebenen haben wird. Zum anderen befürchtet ein beträchtlicher Teil der Befragten aufgrund der stärkeren Belastung der verbliebenen Hausarztpraxen eine Art Negativspirale, wenn es um die Anziehungskraft einer allgemeinärztlichen Tätigkeit aus Sicht junger Mediziner*innen geht (Z3).

Bereits heute konstatieren 53 % der Studienteilnehmer*innen eine stark (27 %) oder eher stark (26 %) sinkende Attraktivität der Hausarztmedizin für den ärztlichen Nachwuchs, wohingegen 36 % von einer stark (5 %) oder eher stark (31 %) steigenden Attraktivität ausgehen. In einer offenen Nachfrage führt ein beträchtlicher Teil der Befragten die grundlegende Problematik eines Imageproblems an, das zu einer Abschreckung nachrückender Ärzt*innen führe (Z4).

Besagtes Attraktivitätsdefizit wird seitens der Befragten schwerpunktmäßig 3 Problembereichen zugeordnet:

- Stellung der hausärztlichen Versorgung im deutschen Gesundheitswesen: Die Befragten monieren eine zu schwach regulierte und daher ineffektive Arbeitsteilung zwischen den Versorgungssektoren. Im Ergebnis führe dies wirtschaftlich, zeit- und ressourcenbezogen unverhältnismäßig stark zu einer Belastung von Hausärzt*innen. Die mangelnde Einbeziehung von Hausärzt*innen im interprofessionellen Zusammenhang führe u. a. zu unnötigen Redundanzen und u. U. zu einer schlechteren Versorgung. Da Nachwuchsmediziner*innen die nachteilige Rolle von hausärztlichen Primärversorger*innen nicht verborgen bleibe, seien diese zumeist nicht geneigt, die Allgemeinmedizin als Arbeitsbereich ins Auge zu fassen.
- Voraussetzungen in Aus- und Weiterbildung: Aufgrund mangelnder Vorbereitung auf eine ambulante und hausärztliche Tätigkeit im Curriculum gehe derzeit nach Dafürhalten vieler Befragter selbst der harte Kern derjenigen, die der Hausarztmedizin zugetan sind, teilweise verloren. Diese Problematik manifestiere sich auch und gerade bei den Themen der Selbstständigkeit und des Praxismanagements. Zudem würden der Wert, der Alltag und die Motivationsfaktoren der hausärztlichen Arbeit nicht im ausreichenden Maße vermittelt.
- Arbeitsbedingungen: Ein weiterer Kritikpunkt richtet sich darauf, dass es die Gesundheitspolitik zu lange versäumt habe, die veränderten Vorstellungen von Berufstätigkeit oder der Vereinbarkeit von Familie und Beruf in Form neuer Beschäftigungsmodelle auszugestalten (z. B. mehr Teilzeitoptionen, flexible Arbeitszeiten, geringerer Wunsch nach Selbstständigkeit, stärkere interdisziplinäre Anbindung).

Ungeachtet des verbreitet skeptischen Ausblicks auf die weitere Entwicklung der hausärztlichen Versorgung würde ein hoher Anteil der Befragten (69 %) Medizinstudierenden oder Ärzt*innen in Weiterbildung heute empfehlen, Hausärzt*in zu werden. Trotz erheblicher Stress- und Belastungsfaktoren im Praxisalltag wird dies mit einer hohen Berufszufriedenheit, intrinsischer Motivation und der Überzeugung begründet, als Hausärzt*in echte Primär- und Breitenversorgung leisten zu können (Z5).

### Persönliche Belastungserfahrungen sowie Beobachtungen zum Ärzt*innenmangel

Ein erheblicher Anteil der konsultierten Allgemeinmediziner*innen beobachtet im eigenen Praxisumfeld einen merklichen Schwund der hausärztlichen Versorgung. So geben 42 % an, die eigene Gegend sei sehr stark (18 %) oder eher stark (24 %) vom Rückgang hausärztlicher Praxen betroffen; 26 % sehen eine geringfügige bis moderate Betroffenheit (27 % kaum bis gar nicht betroffen). Nach Beurteilung von 29 % ist der Rückgang hausärztlicher Praxen inzwischen so groß, dass die entsprechenden Befragten eine beeinträchtigte bzw. erschwerte Versorgungslage in der eigenen Praxisumgebung wahrnehmen; 26 % sehen ein entstandenes, wenn auch noch kein gravierendes Versorgungsdefizit. Ärzt*innen in Kleinstädten und Landgemeinden nehmen gegenüber ihren Kolleg*innen in Mittel- und Großstädten rund doppelt so häufig eine aufgrund von Hausärzt*innenschwund entstandene Mangelversorgung wahr (40 % zu 19 %, *p* < 0,001).

Gefragt nach verschiedenen Belastungsfaktoren für die hausärztliche Tätigkeit, gibt eine überwältigende Mehrheit der Befragten Aspekte wie ein hohes und zeitkonsumierendes Maß an bürokratischen Pflichten sowie empfundenem Kostendruck an, der mitunter Folgen für die Gewährleistung einer optimalen Versorgung hat (Tab. 4). Insbesondere Ärzt*innen in ländlichen Gebieten erleben Schwierigkeiten bei der Suche nach neuem Personal, Folgen der zurückgehenden hausärztlichen Versorgung und die eingeschränkte Verfügbarkeit von Spezialist*innen als belastende Faktoren ihrer Tätigkeit.

**Tab. 4:** Erlebte Problematiken bei der hausärztlichen Arbeit

**Frage:** *Als wie groß erleben Sie die folgenden Problematiken bei der Ausübung Ihrer hausärztlichen Arbeit?* (*N* = 4176; Antwortkategorien „sehr groß“/„eher groß“ wurden zusammengefasst)	Gesamtzustimmung (%)	Urbane vs. Landärzt*innen (%)
Kostendruck und -restriktionen im Gesundheitswesen (z. B. mit Folgen der Einschränkung einer optimalen und individuellen Versorgung)	94	91/97
Bürokratischer Aufwand (z. B. Dokumentations- und Nachweisverpflichtungen)	92	94/90
Schwierigkeiten bei der Personalsuche (z. B. Praxispersonal, angestellte Ärzt*innen)	77	63/91*
Zusätzliche Belastungen aufgrund des Ärztemangels (z. B. weil die eigene Praxis aufgrund anderer Hausarztpraxen, die keine Nachfolge gefunden haben und schließen mussten, mehr Patient*innen versorgen muss)	57	39/75*
Mangelnde Verfügbarkeit von Fachärzt*innen in der Umgebung, um die eigene hausärztliche Arbeit als „Lotse im System“ angemessen ausüben zu können	44	33/55*

Signifikanter Unterschied: *p < 0,001

### Favorisierte Ansätze bzw. Maßnahmen zur Sicherstellung der Hausarztmedizin

Tab. 5 zeigt eine Übersicht der Ansätze und Konzepte, die aus Sicht der Befragten mehr oder weniger effektive Beiträge leisten können, um die hausärztliche Versorgung zu sichern. Erkennbar ist, dass v. a. Maßnahmen favorisiert werden, die die hausärztliche Position im Gesundheitswesen aufwerten und Aus- bzw. Weiterbildung an aktuelle Erfordernisse anpassen. Auffällig hoch ist der Zuspruch in Bezug auf die Einrichtung eines Primärarztsystems als Maßnahme, um – etwa in Kombination mit stärkerer Verbindlichkeit des hausärztlichen Leistungskatalogs – die Handlungsfähigkeit und damit Attraktivität der Hausarztmedizin nachhaltig zu steigern (Z6).

**Tab. 5:** Zustimmung zu Maßnahmen zur Sicherstellung der hausärztlichen Versorgung

**Frage:** *Nachfolgend stehen verschiedene Maßnahmen. Bitte geben Sie jeweils an, für wie effektiv Sie diese Maßnahmen zur längerfristigen Sicherung der hausärztlichen Versorgung halten?* (*N* = 4176; Antwortkategorien „sehr effektiv“/„eher effektiv“ wurden zusammengefasst)	Gesamtzustimmung (%)	Urbane vs. Landärzt*innen (%)
Einführung eines Primärarztsystems, das Hausärzt*innen verbindlich zu ersten Ansprechpartnern für Patient*innen macht und einen direkten und parallelen Besuch von Spezialist*innen vermeidet	85	79/91
Systematische Etablierung ergänzender longitudinaler Begleitprogramme parallel zum Medizinstudium, die Interesse, Einsichten und Kompetenzen in Bezug auf die Hausarztmedizin vermitteln	80	86/74
Verlagerung weg von klassischen Praxismodellen hin zu multiprofessionellen Zentren der ambulanten (Primär‑)Versorgung, um die hausärztliche Versorgung zu erweitern (z. B. Gesundheitszentren in Kliniknähe oder in städtischen Zentren, die multiprofessionelle Kooperation und andere, flexiblere Arbeitsmodelle erlauben)	64	73/55*
Signifikante Verringerung des allgemeinen Kostendrucks für Hausärzt*innen	62	60/64
Deutliche Erhöhung des Anteils der Allgemeinmedizin in der Weiterbildung (z. B. auf ein Drittel)	60	59/61
Inhaltlich-curriculare Umstrukturierung des Medizinstudiums (bessere und gezieltere Vorbereitung auf ambulante, niedergelassene Perspektive)	56	61/51
Grundlegende Reform der allgemeinmedizinischen Weiterbildung (u. a. Verkürzung und Flexibilisierung, stärkere Ausrichtung an den zentralen Kompetenzen für die hausärztliche Arbeit)	53	53/53
Verbindlichkeit des hausärztlichen Leistungskatalogs, um hausärztliche Aufgabenportfolios klar zu umreißen und eine Überlastung von Hausärzt*innen zu verhindern (z. B. durch Sicherstellung von ausreichender Qualifikation und Stundenumfang)	52	48/56
Stärkere Änderung der Zulassungskriterien zum Medizinstudium (in größerer Breite Faktoren wie Persönlichkeit und curriculare Spezifika stärker und umfassender berücksichtigen)	50	52/48
Delegation und verstärkter Einsatz nicht-ärztlicher Gesundheitsberufe sowie Ausweitung von deren Befugnissen	44	32/56*
Effektive ärztliche Personalrekrutierung (verstärkte Arbeit mit Anreizen und Belohnungen, z. B. durch Kommunen und Fördermittel bzw. Prämien, wenn etwa eine Ansiedlung im ländlichen Gebiet erfolgt)	38	30/46*
Grundlegende Aufwertung der Vergütung von Hausärzt*innen (z. B. dass diese mindestens dem Niveau von Spezialist*innen entspricht)	38	36/40
(Stärkere) Bedarfsplanung mit gezielt regionaler Verteilungswirkung	35	29/41
Deutlich mehr Studienplätze im Fach Humanmedizin	35	35/35
Durchgehende, bundesweite Einrichtung einer Landarztquote (für sämtliche Bundesländer klar geregelt, ggf. als On-top-Quote)	34	23/45*
Verstärkter und systematischer Einsatz von Digitalisierung und Telemedizin (u. a. Videosprechstunden, Verschreibung von Gesundheits-Apps zum Selbstmanagement von Patient*innen)	28	43/13*
Quotierung des Zugangs zur Spezialist*innenweiterbildung	25	26/24
Berechtigung zu hausärztlicher Tätigkeit stärker für Quereinsteiger*innen mit anderen disziplinären Hintergründen öffnen	22	25/19

Signifikanter Unterschied: *p < 0,001

Jenseits einer Veränderung des Medizinstudiums (Zulassungskriterien, inhaltliche Neustrukturierung) sprechen sich die Befragten für strukturell fundierte Interventionen im Verlauf des Studiums (longitudinale Begleitprogramme) aus, damit Studierende intensive Einblicke in die hausärztliche Tätigkeit erhalten, interessiert und motiviert werden sowie Unsicherheiten beseitigt und Fertigkeiten frühzeitig erlernt werden können. Zudem sind viele Hausärzt*innen von einer Stärkung der Weiterbildung und dem (verstärkten) Aufbau multiprofessioneller Versorgungszentren überzeugt, nicht zuletzt weil diese als Hebel erachtet werden, um den Beschäftigungsvorstellungen junger Mediziner*innen besser entgegenzukommen (Z7, Z8).

Vergleichsweise wenig Vertrauen zeigen die befragten Ärzt*innen im Hinblick auf Maßnahmen wie Landarztquote, Budgetaufwertungen oder Mittel der Digitalisierung, um die Hausarztmedizin längerfristig zu stabilisieren. Auch die reine Steigerung der Studienplätze wird vom Gros der Befragten nicht per se als hilfreich erachtet (Z9).

Bei den favorisierten Maßnahmen zeigen sich einige hochsignifikante Unterschiede zwischen urbanen und Landärzt*innen. So sprechen sich Erstere in erheblich höherem Maße für Zentren der Primärversorgung aus und sehen deutlich mehr Chancen in digitalen Lösungen. Ärzt*innen mit ländlichem Praxisstandort wiederum erblicken größeren Mehrwert im verstärkten Einsatz von Delegation und nichtärztlichen Gesundheitsberufen, befürworten in stärkerem Maße eine effektive ärztliche Personalrekrutierung zur Ansiedlung in ruralen Gegenden und stehen einer bundesweit einheitlich geregelten Landarztquote erheblich aufgeschlossener gegenüber als urbane Ärzt*innen (Z10, Z11).

Weiter fällt auf, dass Ärzt*innen mit derzeitiger oder früherer Lehrerfahrung im akademisch-allgemeinmedizinischen Kontext im Vergleich zu den übrigen Ärzt*innen deutlich häufiger für die systematische Etablierung ergänzender longitudinaler Begleitprogramme parallel zum Medizinstudium plädieren (94 % zu 71 %, *p* < 0,001). Ähnliches gilt für die inhaltlich-curriculare Umstrukturierung des Medizinstudiums und eine stärkere Änderung der Zulassungskriterien zum Medizinstudium (Z12).

### Beurteilung ergriffener Maßnahmen zur Sicherstellung und weitere Optimierungsansätze

Während 36 % mit den bislang ergriffenen gesundheitspolitischen Anstrengungen zur Stabilisierung der hausärztlichen Versorgung sehr (4 %) oder eher zufrieden (32 %) sind, zeigen sich 64 % sehr (18 %) oder eher (46 %) unzufrieden. Zwar wird in den offenen Antworten immer wieder auf zuletzt wieder angestiegene fachärztliche Anerkennungszahlen hingewiesen, allerdings seien diese immer noch deutlich zu niedrig, um den absehbaren Bedarf an nachrückenden hausärztlichen Versorger*innen zu decken (Z13, Z14).

Jenseits limitierter Ressourcen, die von gesundheitspolitischen Akteuren zur Stützung der Primärversorgung aufgewandt werden, bündelt sich die Kritik der Befragten in verschiedenen Schwerpunktbereichen. Dies betrifft zunächst die inhaltliche Ausgestaltung und Steuerung im Studienverlauf, der deutlich mehr auf die ambulante Versorgung ausgerichtet werden müsse. Die mit dem Masterplan 2020 initiierte Reform des Humanmedizinstudiums, so führt eine Reihe von Befragten an, habe nur zaghafte curriculare Neujustierungen vorgenommen und sei bis heute nicht konsequent umgesetzt (Z15, Z16).

In Bezug auf die Landarztquote äußern sich auffallend viele Befragte zurückhaltend und sehen in der bisherigen Ausgestaltung kaum positive Effekte für substanziell steigende Zahlen nachrückender Hausärzt*innen. Dies wird maßgeblich darauf zurückgeführt, dass viele Bundesländer keine On-top-Quoten für die teils eingerichtete Landarztquote beschlossen haben, sondern einen Anteil der bestehenden Studienplätze hierfür vorsehen. Auch dass die Ausgestaltung der Landarztquote im föderalen Gefüge stark uneinheitlich ist, wird kritisiert. Viele Hausärzt*innen halten es für gewinnbringender, mehr Anstrengungen bei der Suche und Auswahl nach künftigen Allgemeinärzt*innen aufzuwenden (Z17, Z18).

Während beim Medizinstudium immerhin Reformbemühungen erkennbar seien, bestehen aus Sicht der Befragten im Bereich der Weiterbildung nach wie vor zu starke Beharrungskräfte und ein entsprechender Reformstau (Z19).

Ein wertvoller Hebel, um das Heranziehen von Hausärzt*innen aus der Weiterbildung heraus zu fördern, wird von einem Teil der Befragten in den nahezu in allen Bundesländern eingerichteten Kompetenzzentren Allgemeinmedizin gesehen, die die Weiterbildung aktiv unterstützen (Z20, Z21).

Ferner, so wünschen sich viele Befragte, dürfe die Hausarztmedizin nicht mehr unter dem ständigen Druck und Einfluss von Kostenrestriktionen stehen; vielmehr müsse ihre Eigenständigkeit und Souveränität im Gesundheitswesen gestärkt werden. Die Einrichtung eines Primärarztsystems im Sinne eines strukturierten hausarztzentrierten Modells wird in diesem Zusammenhang erneut von einem Teil der Ärzt*innen herausgestellt (Z22).

Wenn es darum geht, einem zukünftigen Hausärztemangel vorzubeugen, wünscht sich eine Mehrheit der befragten Allgemeinmediziner*innen (62 %) eine stärkere Einbindung ihrer Berufsvertreter*innen in gesundheitspolitisch relevante Gremien, damit sie ihre Perspektive besser einbringen und Maßnahmen entsprechend mitgestalten können. In der Folgefrage konnten die Befragten zwischen 3 Ansätzen wählen, mittels derer die Perspektive von Hausärzt*innen institutionell besser verankert werden könnte. 27 % entschieden sich für eine Aufstockung der hausärztlichen Besetzung in ärztlichen und wissenschaftsnahen Gremien mit gesundheitspolitischem Bezug, während 24 % ein stärkeres und systematischeres Herantreten an Akteure der Gesundheitspolitik von Bund, Ländern und Kommunen aus den organisierten Verbänden bzw. Fachgesellschaften heraus (z. B. Hausärzteverband, Deutsche Gesellschaft für Allgemeinmedizin und Familienmedizin – DEGAM) favorisieren. 40 % sprechen sich für den Vorschlag des Deutschen Ärztetages aus, der 2023 die Einrichtung eines ressortübergreifenden Deutschen Gesundheitsrats unter Beteiligung der Bundesärztekammer (BÄK) und weiterer Vertreter der Selbstverwaltung sowie der Wissenschaft forderte. Ähnlich dem Deutschen Ethikrat soll sich der Deutsche Gesundheitsrat proaktiv oder im Auftrag entsprechender Fachressorts in politische Prozesse einbringen [26].

Trotz aktuell bestehender Sorgen in Bezug auf die Stabilisierung der ambulanten Breitenversorgung erachten die Befragten das hausärztliche Berufsbild gerade in Zeiten zunehmender disziplinärer Spezialisierung als wichtiger denn je. Gelinge es, das hausärztliche Metier durch wirksame Maßnahmen zu stärken und neu zu vitalisieren, so werde dies das Gesundheitswesen insgesamt absichern (Z23, Z24).

## Diskussion

Um in Zukunft auf ein leistungsfähiges und resilientes Gesundheitssystem zurückgreifen zu können, kommt der hausärztlichen Primärversorgung eine Schlüsselrolle zu [27]. Wie die Ergebnisse der vorliegenden Befragung von 4176 Allgemeinmediziner*innen untermauern, ist die Problematik eines Schwundes hausärztlicher Versorger*innen in der Mitte der Hausärzteschaft angekommen. Ein erheblicher Teil der Befragten erlebt inzwischen Belastungen und Engpässe aufgrund des sich regional teils merklich ausdünnenden Versorgungsnetzes. Hierbei decken sich die Befunde zum Erleben schwindender Versorgung mit dem MLP-Gesundheitsreport 2022, einer repräsentativen Trendbefragung von Ärzt*innen [23].

Laut den Ergebnissen der vorliegenden Arbeit blicken viele Allgemeinmediziner*innen sorgenvoll in die Zukunft, wenn es um die langfristige Gewährleistung der hausärztlichen Versorgung geht. Trotz partieller Fortschritte bei der Stärkung der Hausarztmedizin wird großer Handlungsbedarf für die nächsten Jahre ausgemacht, um das Fachgebiet für den medizinischen Nachwuchs attraktiver zu machen. Die größten Probleme stellen nach Dafürhalten der Befragten strukturelle Problematiken des deutschen Gesundheitssystems, aber auch ein Stau notwendiger Reformen in der Aus- und Weiterbildung dar. Hinzu kommen u. a. Defizite von (regionaler) Bedarfsplanung und Anreizförderung.

### Gegenüberstellung mit Befunden anderer Studien

In Bezug auf die von Hausärzt*innen favorisierten Maßnahmen, die in dieser Studie anhand einer großen Stichprobe breit abgefragt werden konnten, ähneln die Befunde stark den Ergebnissen der vorangegangenen qualitativen Interviewstudie mit Allgemeinärzt*innen [20]. Dabei sind mit Blick auf bestimmte Lösungsansätze zur Sicherung der allgemeinärztlichen Versorgung zunächst Gemeinsamkeiten zwischen den Antworten der Hausärzt*innen und den Vorschlägen von Expert*innen der Versorgungsforschung oder dem „Sachverständigenrat zur Begutachtung der Entwicklung im Gesundheitswesen“ [28] erkennbar. Jenseits der Verringerung von Kostendruck und -restriktionen [22, 29] sieht das Gros der Befragten v. a. die Einrichtung eines Primärarztsystems als wirksames Konzept [13]. Dieses wird bereits seit Jahren von der DEGAM gefordert, um die Hausarztmedizin im Gesundheitswesen zu repositionieren und günstigere Voraussetzungen für seine Gewährleistung zu schaffen [12].

Auch das Gutachten des Sachverständigenrats (SVR) fordert verstärkte Schritte in Richtung einer hausarztzentrierten Versorgung [27]. Begründet wird dies nicht nur mit einer gesteigerten Attraktivität der Hausarztmedizin, sondern auch mit der bestehenden Evidenz über ihren Versorgungsmehrwert (u. a. niedrigere 5‑Jahres-Mortalität, geringeres Hospitalisierungsrisiko, erhöhte Versorgungskontinuität; [30–33]). Um eine weitergehende Hausarztzentrierung zu erreichen, so der SVR, müsse die hausärztliche Versorgung internationalen Vorbildern folgend weiter optimiert werden. Hierfür werden allerdings deutlich mehr und entsprechend qualifizierte Fachärzt*innen für Allgemeinmedizin benötigt.

Weitere Schnittmengen bestehen zwischen den befragten Hausärzt*innen und den Forderungen von Expert*innen hinsichtlich einer möglichst verbindlichen Definition des hausärztlichen Leistungskatalogs sowie der Etablierung und kontinuierlichen Förderung multiprofessioneller Versorgungszentren (Chance für Synergiepotenziale mit sektorenübergreifenden Lösungen; [5, 34]). Ähnliches gilt für den Ausbau von Delegationskonzepten (Einsatz nichtärztlicher Gesundheitsberufe; [14, 35]), aber auch bezüglich einer stärkeren Regulierung des Zugangs zur fachärztlichen Weiterbildung, welche stärker am allgemeinen Bedarf ausgestaltet werden sollte [13, 18].

Gleichzeitig zeigen die Befragungsresultate, dass es von Bedeutung ist, die Sichtweisen von Allgemeinmediziner*innen zur Thematik einzuholen, da sie eigene Schwerpunkte und Prioritäten setzen, die sich mitunter von bislang verfügbaren Expertisen unterscheiden. Besonders deutlich wird dies etwa mit Blick auf longitudinale Interventionen im Studienverlauf, die von den Befragten als hochwirksam erachtet werden, z. B. bei Studierenden bereits zu einem frühen Zeitpunkt Interesse an der Hausarztmedizin zu wecken sowie Kenntnisse und Kompetenzen für die niedergelassene Tätigkeit zu vermitteln. Im Masterplan 2020 ist der längsschnittliche Unterricht durch die Allgemeinmedizin als ein Kernelement angelegt [36]. Auch hat der SVR in seinem Gutachten den Stellenwert von additionalen Angeboten im allgemeinmedizinischen Studium hervorgehoben, darunter freiwillige Landarzt-Tracks oder eine verstärkte Berufsfelderkundung [28]. Tatsächlich konnten internationale Arbeiten nachweisen, dass sich die Teilnahme an allgemeinmedizinischen Blockpraktika signifikant auf die Berufspräferenz bzw. Niederlassungsbereitschaft auswirken kann [22, 37, 38]. Den Autoren ist im Zuge ihrer Recherchen aufgefallen, dass im deutschsprachigen Raum präsente Autor*innen, wie z. B. van den Bussche et al. [13, 18, 19], die Thematik der longitudinalen Intervention nicht im gleichen Maße prominent behandeln wie andere Forderungen zur Reform der Hausarztmedizin.

Viele Allgemeinmediziner*innen halten es für entscheidend, dass klassische Arbeits- und Beschäftigungsmodelle in moderne Formen überführt werden, die nachrückenden Generationen entgegenkommen. Daher ist bezeichnend, dass sich eine vergleichsweise große Gruppe zugunsten neuer, integrativer Settings wie Primärversorgungszentren ausspricht, die von der klassischen Einzelpraxis wegführen [39–41]. Diesbezüglich gibt es Schnittmengen mit den Studien von Huenges et al. [42] oder auch van den Bussche et al. [13, 19, 43], die Ärzt*innen in Weiterbildung befragt haben. In besagten Studien zeigte sich der Bedarf an einer weiterreichenden Reform der Weiterbildung mit Blick auf Dauer, Inhalte und Didaktik. Kompetenzzentren, die auch der SVR als flächendeckende, universitär angebundene Einrichtungen angeregt hat [28], können als „natürliche“ Partner in Anknüpfung an das Studium eine wirksame Begleitung leisten. So können sie flankierend die Weiterbildung abstützen und mittels strukturierter Kursprogramme, Mentoring oder Aufbau von Weiterbildungsverbünden zu einer Stärkung des hausärztlichen Nachwuchses beitragen [18]. Nicht zuletzt können sie Abbruchraten in der Weiterbildung verringern helfen [44].

Weiter fällt auf, dass Hausärzt*innen bestimmte in der Öffentlichkeit präsente und auch im gesundheitspolitischen Diskurs diskutierte Ansätze nur als begrenzt hilfreich empfinden. Dies gilt etwa für die Landarztquote, der viele Befragte keinen großen Nutzen unterstellen. So wird Kritik an der bundeslandspezifischen Ausgestaltung und den quantitativ stark begrenzten Kapazitäten des bislang ergriffenen Landarztquotenmodells geübt. Darüber hinaus zeigte die Vorstudie, dass Hausärzt*innen die Befürchtung haben, das Quotenprinzip könnte einen defizitären Zustand der hausärztlichen Versorgung in der Öffentlichkeit unterstreichen [20]. Im Kontrast zu vorgelegten Expertisen sind die Befragten reserviert, wenn es darum geht, Quereinsteiger*innen stärker für die Hausarztmedizin zuzulassen und die Hürden der Weiterbildung zu senken [12, 13, 45].

Entsprechend des artikulierten Wunsches der Befragten sollten Hausärzt*innen bei der Planung, Implementierung und Evaluation von Maßnahmen zur Bekämpfung des (drohenden) Mangels von Primärversorger*innen konsequent einbezogen werden. In ärztlichen und wissenschaftsnahen Gremien ist ihre Berufsgruppe zumeist in der Minderheit und sollte daher verstärkt durch die gesundheitspolitischen Entscheidungsträger*innen berücksichtigt werden.

### Stärken und Schwächen

Die Befragung hat auf einer qualitativen Vorstudie aufgebaut und war daher konkret und praxisnah auf die hausärztliche Perspektive zugeschnitten. Eine Reihe offener Fragen ermöglicht die Erfassung nichtstandardisierter Informationen. Zudem erzielte die Befragung einen vergleichsweise hohen Rücklauf, sodass die erreichte Stichprobe im Hinblick auf Merkmale und Einstellungen in Bezug auf die untersuchte Thematik breit gestreut ist.

Dennoch ist auf diverse Limitationen hinzuweisen. So kann die Studie nicht im strengen Sinne, sondern – durch einen Vergleich mit KV-Daten – höchstens angenähert einen repräsentativen Anspruch erheben (begrenzte Fallzahl, regionale Rekrutierung). Zudem ist es möglich, dass Ärzt*innen mit Interesse an der untersuchten Thematik sich stärker an der Studie beteiligt haben.

Auch ist darauf hinzuweisen, dass es sich um Standpunkte und Meinungen der befragten Hausärzt*innen mit Blick auf die Sicherstellung der allgemeinärztlichen Versorgung handelt. Die Autoren erachten die vorliegende Arbeit als Ergänzung von Studien, die sich etwa mit Karriereverläufen bzw. -brüchen angehender Mediziner*innen in Studium und Weiterbildung befasst haben. Hier sind ausschließlich bereits in der Versorgung tätige Ärzt*innen befragt worden, was eine spezielle Sicht mit Blick auf die untersuchte Thematik bedeutet.

Aufgrund der Notwendigkeit, eine kompakte Befragung zu erstellen, mussten bei der Ausgestaltung des Instruments Prioritäten gesetzt werden. Eine (verstärkte) Erhebung von Fragen zur persönlichen Motivation für die eigene Berufs‑/Fachwahl oder zu den Vorzügen der hausärztlichen Tätigkeit wäre von Interesse gewesen, konnte aber nicht realisiert werden. Ähnliches gilt für die Erhebung individueller Maßnahmen, wie einzelne Hausärzt*innen einen Beitrag zur Verbesserung der Attraktivität der Hausarztmedizin leisten könnten. Dies wären Themen, die eine Folgestudie verstärkt in den Blick nehmen könnte.

### Schlussfolgerungen

Hausärzt*innen sehen großen Handlungsbedarf, um einen zu starken Schwund allgemeinärztlicher Versorger*innen abzuwenden. Dies geht einher mit einer von vielen Befragten beobachteten Verschärfung der Versorgungslage vor Ort. Dieser Problematik zugrunde liegt aus Sicht der Befragten ein umfassendes Attraktivitätsproblem, das zu einem zu geringen Zufluss nachrückender Ärzt*innen in die Hausarztmedizin führt. Als wirksamen Ansatz zur Stärkung der Primärversorgung regen die in die Studie einbezogenen Hausärzt*innen v. a. die Schaffung eines Primärarztsystems bzw. eine Aufwertung der Rolle hausärztlicher Versorger*innen an. Zudem wird eine stärkere Förderung von Interesse und Berührungspunkten in Bezug auf die Hausarztmedizin in Aus- und Weiterbildung für sinnvoll erachtet, darüber hinaus eine Restrukturierung von Curricula und Zulassungskriterien zum Medizinstudium sowie eine Reform und Stärkung der allgemeinmedizinischen Weiterbildung. Nicht zuletzt wird eine stärkere Überführung der klassischen Hausarztmedizin in multiprofessionelle Zentren als wertvoller Beitrag angesehen.

## Fazit

Die Sicherstellung der hausärztlichen Versorgung konfrontiert die handelnde Gesundheitspolitik mit komplexen Herausforderungen. Viele Allgemeinmediziner*innen blicken sorgenvoll in die Zukunft, wenn es um die langfristige Gewährleistung der hausärztlichen Versorgung geht. Trotz partieller Fortschritte wird großer Handlungsbedarf für die nächsten Jahre ausgemacht. Hausärzt*innen benennen 3 Bereiche, über die die Attraktivität der Hausarztmedizin für ärztlichen Nachwuchs spürbar erhöht werden kann: 1) Stellung der hausärztlichen Versorgung im Gesundheitswesen, 2) Voraussetzungen in Aus- und Weiterbildung, 3) Arbeitsbedingungen. Entsprechend plädieren die Befragten v. a. für die Einrichtung eines Primärarztsystems, die Förderung von Interesse und Berührungspunkten im Studium (longitudinale Begleitprogramme) sowie den Ausbau multiprofessioneller ambulanter Versorgungszentren.

### Supplementary Information

Fragebogen zur Studie
