# Supplementary material for: Ensuring primary care in Germany—findings from a quantitative survey of general practitioners
Source: Bundesgesundheitsblatt Gesundheitsforschung Gesundheitsschutz. 2024 Jun 11;67(9):998–1009. [Article in German] doi: 10.1007/s00103-024-03896-4 (PMC11349858; doi:10.1007/s00103-024-03896-4)
Supplement: Supplementary file 1 — Fragebogen zur Studie [file 103_2024_3896_MOESM1_ESM.pdf]

1. Wie schätzen Sie das ein: Wird sich die Gesundheitsversorgung in Deutschland in den nächsten Jahren insgesamt eher verbessern oder eher verschlechtern?

◯ Deutlich verbessern ◯ Etwas verbessern ◯ Gleich bleiben ◯ Etwas verschlechtern ◯ Deutlich verschlechtern

◯ Schwer zu sagen, weiß nicht

2. Und wie schätzen Sie dies für speziell für die ambulante Versorgung ein, also niedergelassene Haus- und Fachärzt*innen? Wird sich die Gesundheitsversorgung hier…

◯ Deutlich verbessern ◯ Etwas verbessern ◯ Gleich bleiben ◯ Etwas verschlechtern ◯ Deutlich verschlechtern

◯ Schwer zu sagen, weiß nicht

3. Beobachten Sie zurzeit eine eher steigende oder eher sinkende Attraktivität der Hausarztmedizin für ärztlichen Nachwuchs, wenn Sie dies mit der Situation vor einigen Jahren vergleichen?

◯ Stark steigend ◯ Eher steigend ◯ Eher sinkend ◯ Stark sinkend

◯ Schwer zu sagen, weiß nicht

4. Worauf führen Sie dies zurück? Formulieren Sie Ihre Antwort gerne anhand von Schlagworten und Stichpunkten.

____

____

____

5. Würden Sie Medizinstudierenden oder Ärzt*innen in der Weiterbildung heute empfehlen, Hausärzt*in zu werden oder würden Sie eher davon abraten?

◯ Voll und ganz empfehlen ◯ Eher empfehlen ◯ Eher abraten ◯ Voll und ganz abraten

◯ Schwer zu sagen, weiß nicht

6. Warum vertreten Sie diese Auffassung? Formulieren Sie Ihre Antwort gerne anhand von Schlagworten und Stichpunkten.

____

____

____

7. Als wie groß erleben Sie die folgenden Problematiken bei der Ausübung Ihrer hausärztlichen Arbeit?

	Sehr groß	Eher groß	Weniger bzw. gar nicht groß	Schwer zu sagen
Bürokratischer Aufwand (z.B. Dokumentations- und Nachweisverpflichtungen)	◯	◯	◯	◯
Schwierigkeiten bei der Personalsuche (z.B. Praxispersonal, angestellte Ärzt*innen)	◯	◯	◯	◯
Kostendruck und -restriktionen im Gesundheitswesen (z.B. mit Folgen der Einschränkung einer optimalen und individuellen Versorgung)	◯	◯	◯	◯
zusätzliche Belastungen aufgrund des Ärztemangels (z.B. weil die eigene Praxis aufgrund anderer Haus-arztpraxen, die keine Nachfolge gefunden haben und schließen mussten, mehr Patienten versorgen muss)	◯	◯	◯	◯
Mangelnde Verfügbarkeit von Fachärzt*innen in der Umgebung, um die eigene hausärztliche Arbeit als ‚Lotse im System' angemessen ausüben zu können	◯	◯	◯	◯
Sonstiges, und zwar:	◯	◯	◯	◯

8. Wenn es um Zukunft und Entwicklungsperspektiven der hausärztlichen Versorgung im Zeitraum der nächsten 10 bis 20 Jahre geht: Sind Sie diesbezüglich eher zuversichtlich oder eher besorgt?

◯ Sehr zuversichtlich ◯ Eher zuversichtlich ◯ Eher besorgt ◯ Sehr besorgt

◯ Schwer zu sagen, weiß nicht

9. Welche größeren Veränderungen sehen Sie auf die hausärztliche Versorgung zukommen? Formulieren Sie Ihre Antwort gerne anhand von Schlagworten und Stichpunkten.

____

____

____

10. Wenn es um die längerfristige Gewährleistung der hausärztlichen Versorgung geht. Wie schätzen Sie dies ein: Wie gut ist die hausärztliche Versorgung in Deutschland für die kommenden Jahrzehnte gesichert?

◯ Sehr gut gesichert ◯ Eher gut gesichert ◯ Eher nicht so gut gesichert ◯ Überhaupt nicht gesichert

◯ Schwer zu sagen, weiß nicht

11. Wo bestehen Ihrer Ansicht nach die größten Herausforderungen und Probleme, wenn es um die längerfristige Gewährleistung der hausärztlichen Versorgung geht? Formulieren Sie Ihre Antwort gerne anhand von Schlagworten und Stichpunkten.

____

____

____

12. a) Sind Sie selbst Praxisinhaber*in

◯ Ja ◯ Nein => Bitte direkt **übergehen zu Frage 13**

12. b) Einmal angenommen, Sie würden sich dazu entschließen, Ihre Praxis aufzugeben: Was erwarten Sie, wie schwierig oder leicht wäre es in diesem Fall, eine Nachfolge zu finden?

◯ Sehr schwierig ◯ Eher schwierig ◯ Eher leicht ◯ Sehr leicht

◯ Schwer zu sagen, weiß nicht

13. a) Was würden Sie sagen: Wie stark ist die Gegend, in der Ihre Hausarztpraxis liegt, von einem Schwund an hausärztlicher Versorgung bzw. einem Rückgang hausärztlicher Praxen betroffen?

◯ Sehr stark betroffen ◯ Eher stark betroffen ◯ Etwas betroffen

◯ Geringfügig bis gar nicht betroffen => Bitte direkt **übergehen zu Frage 14** ◯ Schwer zu sagen, weiß nicht

13. b) Und würden Sie sagen, dass es aufgrund des von Ihnen angebenden Schwundes der hausärztlichen Versorgung in Ihrer Gegend bereits spürbare Anzeichen einer Mangelversorgung gibt (z.B. weil die verbliebenen Praxen Probleme haben, die zusätzlichen Patient*innen aufzunehmen) oder ist dies derzeit nicht der Fall?

◯ Mangelversorgung deutlich spürbar ◯ Mangelversorgung ist etwas spürbar

◯ Mangelversorgung derzeit nicht spürbar ◯ Schwer zu sagen, weiß nicht

14. Wenn Sie sich in die Situation der hausärztlichen Versorgung in 10 bis 20 Jahren versetzen: Sehen Sie hier, bezogen auf das Versorgungsangebot an Hausärzt*innen in Deutschland, einen erheblichen breitflächigen Mangel an Hausärzt*innen, oder sehen Sie einen Mangel vorwiegend in ländlichen und strukturschwachen Regionen, oder sehen Sie eher keinen nennenswerten Mangel an Hausärzt*innen? (Bitte nur eine Antwortoption auswählen.)

◯ Erheblicher, breitflächiger Mangel an Hausärzt*innen

◯ (Verstärkter) Mangel an Hausärzt*innen in ländlichen und strukturschwachen Regionen

◯ Kein nennenswerter Mangel an Hausärzt*innen => Bitte direkt **übergehen zu Frage 16**

◯ Schwer zu sagen, weiß nicht

15. Bezogen auf den gesamten Versorgungsbedarf: Wie viel Prozent an Hausärzt*innen werden in 10 bis 20 Jahren in Deutschland fehlen, was würden Sie grob geschätzt vermuten?

◯ 5 bis 10 Prozent ◯ 10 bis 15 Prozent ◯ 15 bis 20 Prozent ◯ Mehr als 20 Prozent

◯ Schwer zu sagen, weiß nicht

16. Welche Voraussetzungen und Maßnahmen wären aus Ihrer Sicht besonders vielversprechend und sollten vordringlich ergriffen werden, um die hausärztliche Versorgung längerfristig zu gewährleisten? Formulieren Sie Ihre Antwort gerne anhand von Schlagworten und Stichpunkten.

____

____

____

17. Nachfolgend stehen verschiedene Maßnahmen. Bitte geben Sie jeweils an, für wie effektiv Sie diese Maßnahmen zur längerfristigen Sicherung der hausärztlichen Versorgung halten?

	Sehr effektiv	Eher effektiv	Weniger bzw. gar nicht effektiv	Schwer zu sagen
Deutlich mehr Studienplätze im Fach Humanmedizin	◯	◯	◯	◯
Stärkere Änderung der Zulassungskriterien zum Medizinstudium (in größerer Breite Faktoren wie Persönlichkeit und curriculare Spezifika stärker und umfassender berücksichtigen)	◯	◯	◯	◯
Inhaltlich-curriculare Umstrukturierung des Medizinstudiums (bessere und gezieltere Vorbereitung auf ambulante, niedergelassene Perspektive)	◯	◯	◯	◯

Systematische Etablierung ergänzender longitudinaler Begleitprogramme parallel zum Medizinstudium, die Interesse, Einsichten und Kompetenzen in Bezug auf die Hausarztmedizin vermitteln	○	○	○	○
Durchgehende, bundesweite Einrichtung einer Landarztquote (für sämtliche Bundesländer klar geregelt, ggf. als On-top-Quote)	○	○	○	○
Grundlegende Reform der allgemeinmedizinischen Weiterbildung (u.a. Verkürzung und Flexibilisierung, stärkere Ausrichtung an den zentralen Kompetenzen für die hausärztliche Arbeit)	○	○	○	○
Effektive ärztliche Personalrekrutierung (verstärkte Arbeit mit Anreizen und Belohnungen, z.B. durch Kommunen und Fördermittel bzw. Prämien, wenn etwa eine Ansiedlung im ländlichen Gebiet erfolgt)	○	○	○	○
(Stärkere) Bedarfsplanung mit gezielt regionaler Verteilungswirkung	○	○	○	○
Deutliche Erhöhung des Anteils der Allgemein-medizin in der Weiterbildung (z.B. auf ein Drittel)	○	○	○	○
Quotierung des Zugangs zur Spezialist*innenweiterbildung	○	○	○	○
Berechtigung zu hausärztlicher Tätigkeit stärker für Quereinsteiger*innen mit anderen disziplinären Hintergründen öffnen	○	○	○	○
Verbindlichkeit des hausärztlichen Leistungskatalogs, um hausärztliche Aufgabenportfolios klar zu umreißen und eine Überlastung von Hausärzt*innen zu verhindern (z.B. durch Sicherstellung von ausreichender Qualifikation und Stundenumfang)	○	○	○	○
Signifikante Verringerung des allgemeinen Kostendrucks für Hausärzt*innen	○	○	○	○
Grundlegende Aufwertung der Vergütung von Hausärzt*innen (z.B. dass diese mindestens dem Niveau von Spezialist*innen entspricht)	○	○	○	○
Einführung eines Primärarztsystems, das Hausärzt*innen verbindlich zu ersten Ansprech-partnern für Patient*innen macht und einen direkten und parallelen Besuch von Spezialist*innen vermeidet	○	○	○	○
Verlagerung weg von klassischen Praxismodellen hin zu multiprofessionellen Zentren der ambulanten (Primär-)Versorgung, um die hausärztliche Versorgung zur erweitern (z.B. Gesundheitszentren in Kliniknähe oder in städtischen Zentren, die multiprofessionelle Kooperation und andere, flexiblere Arbeitsmodelle erlauben)	○	○	○	○
Delegation und verstärkter Einsatz nicht-ärztlicher Gesundheitsberufe sowie Ausweitung von deren Befugnissen	○	○	○	○

Verstärkter und systematischer Einsatz von Digitalisierung und Telemedizin (u.a. Videosprechstunden, Verschreibung von Gesundheits-Apps zum Selbstmanagement von Patient*innen)	◯	◯	◯	◯
Sonstiges, und zwar:	◯	◯	◯	◯

18. Wenn es eine Maßnahme zur langfristigen Sicherung der Hausarztmedizin gibt, die Sie am meisten favorisieren und für besonders vordringlich halten: Welche Maßnahme ist dies?

____

19. In den vergangenen Jahren wurden von der Gesundheitspolitik verschiedene Anstrengungen unternommen, um die hausärztliche Versorgung zu stützen und sicherzustellen. Wie zufrieden sind Sie mit der Gesamtheit dieser bislang ergriffenen Maßnahmen? (Bitte versuchen Sie, Ihren allgemeinen Eindruck bzw. Ihre Beobachtungen hierzu zu verdichten.)

◯ Sehr zufrieden ◯ Eher zufrieden ◯ Eher unzufrieden ◯ Sehr unzufrieden

◯ Schwer zu sagen, weiß nicht

20. Warum sind Sie zufrieden bzw. unzufrieden, womit hängt dies maßgeblich zusammen?

____

____

____

21. Wo müssten die Anstrengungen Ihrer Auffassung nach besonders verstärkt oder verändert werden, damit die hausärztliche Versorgung längerfristig gesichert werden kann? Formulieren Sie Ihre Antwort gerne anhand von Schlagworten und Stichpunkten

____

____

____

22. Wenn es darum geht, einen möglichen Hausärztemangel in der Zukunft zu bekämpfen: Sind Hausärzt*innen Ihres Erachtens ausreichend in gesundheitspolitisch relevanten Gremien berücksichtigt, sodass sie ihre Perspektive einbringen und Maßnahmen mitgestalten können, oder besteht hier noch Nachholbedarf?

◯ Ausreichend vertreten ◯ Sehe gewissen Nachholbedarf ◯ Sehe großen Nachholbedarf

◯ Schwer zu sagen, weiß nicht

23. Wie könnte die Perspektive von Hausärzt*innen bei der Entscheidung über Maßnahmen zur Bekämpfung eines Hausärztemangels besser zur Geltung kommen? Bitte wählen Sie den Punkt aus, der Ihnen am vielversprechendsten erscheint. (Bitte nur eine Antwortoption auswählen.)

◯ Aufstockung der hausärztlichen Besetzung in ärztlichen und wissenschaftsnahen Gremien mit gesundheitspolitischem Bezug bzw. Nähe zu gesundheitspolitischen Entscheidungsträger*innen

◯ Stärkeres und systematischeres Herantreten an Akteure der Gesundheitspolitik von Bund, Ländern und Kommunen aus den organisierten Verbänden bzw. Fachgesellschaften heraus (z.B. Hausärzteverband, DEGAM)

◯ Vorschlag des Deutschen Ärztetages (2023): Einrichtung eines ressortübergreifenden Deutschen Gesundheitsrats unter Beteiligung der Bundesärztekammer (BÄK) und weiterer Vertreter der Selbstverwaltung sowie der Wissenschaft. Hierbei würden auch Hausärzt*inenn berücksichtigt. Ähnlich dem Deutschen Ethikrat soll sich der Deutsche Gesundheitsrat proaktiv oder im Auftrag entsprechender Fachressorts in politische Prozesse einbringen.

◯ Sonstiges, und zwar:

____

____

Nun bräuchten wir noch einige statistische Angaben von Ihnen. Wie auch der Rest des Fragebogens werden diese Informationen natürlich streng vertraulich behandelt und anonymisiert.

Sind sind…

◯ Männlich ◯ Weiblich ◯ Divers

Ihr **Alter**: _____

Wo befindet sich Ihre Praxis? In einer Gemeinde/Stadt mit . . .

◯ mehr als 100.000 ◯ 20.000 bis 100.000 ◯ 5.000 bis 20.000 ◯ unter 5.000 Einwohner*innen

Welches Niederlassungsmodell beschreibt Ihre Praxis am besten?

◯ Einzelpraxis (Sie sind der/die einzige Arzt/Ärztin) ◯ Einzelpraxis mit angestellten Ärzt*innen

◯ Gemeinschaftspraxis ◯ Medizinisches Versorgungszentrum ◯ Sonstiges

Wie viele Patient*innen behandelt Ihre Praxis im Quartal?

◯ 500 bis 750 ◯ 751 bis 1000 ◯ 1001 bis 1500 ◯ 1501 bis 2000 ◯ Mehr als 2000

Waren oder sind Sie als **akademische/r Lehrarzt/-ärztin** tätig, haben also bereits bei der Ausbildung von Medizinstudierenden Aufgaben übernommen (z.B. Praktika, Famulatur, Blockpraktikum Allgemeinmedizin, Übernahme von Lehrveranstaltungen)?

◯ Ja, aktuell als Lehrarzt/-ärztin tätig ◯ Ja, früher als Lehrarzt/-ärztin tätig ◯ Nein

Vielen Dank für Ihre Teilnahme!

Was möchten Sie uns noch mitteilen? Hier finden Sie Platz für Anregungen, Kommentare und Kritik.

____

____

____
